# Supplementary figures and images for: CIAlign: A highly customisable command line tool to clean, interpret and visualise multiple sequence alignments
Source: PeerJ. 2022 Mar 15;10:e12983. doi: 10.7717/peerj.12983 (PMC8932311; doi:10.7717/peerj.12983)

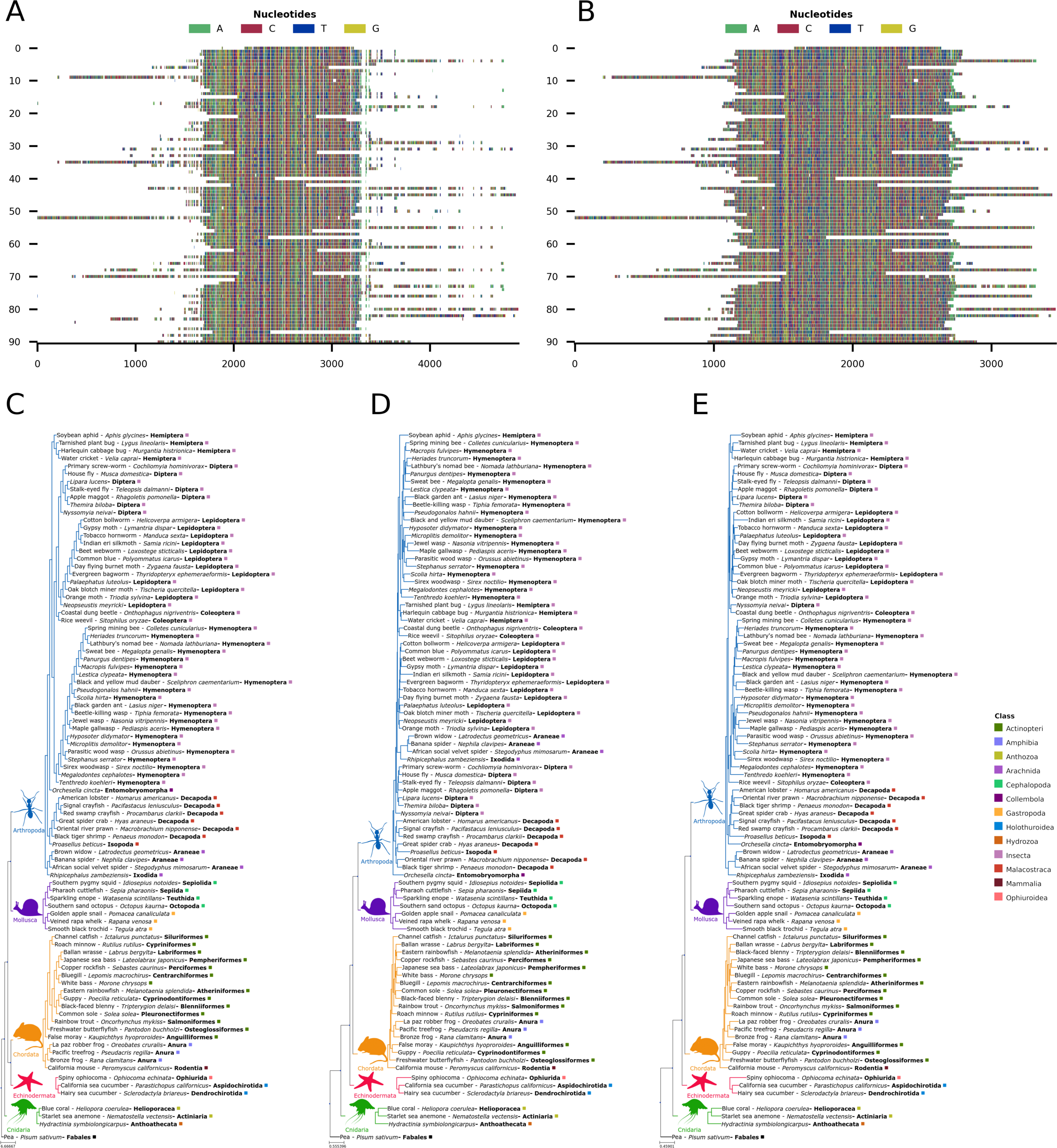

Supplement: Supplemental Information 1 — (A) Input alignment before application of CIAlign, generated using the command “CIAlign infile example4.fastaplot_input”. (B) Output alignment after application of CIAlign, generated using the command “CIAlign infile example4.fastaall remove_divergent_minperc 0.5”. Subplots were generated using the “draw mini alignment function. (C) Phylogenetic tree generated manually using the literature to show the current best estimate for the phylogenetic relationships between these 91 families of metazoa. Relationships are based on the literature listed in the Supp. References. (D) PhyML (Smith, 2019) phylogenetic tree generated under the GTR model plus default settings on the input alignment before application of CIAlign. (E) PhyML (Smith, 2019) phylogenetic tree generated under the GTR model plus default settings on the cleaned alignment after application of CIAlign. In (C–E) branch colours correspond to the labelled phyla, coloured squares indicate class and bold text indicates order. Common names are shown where available. [file peerj-10-12983-s001.png]

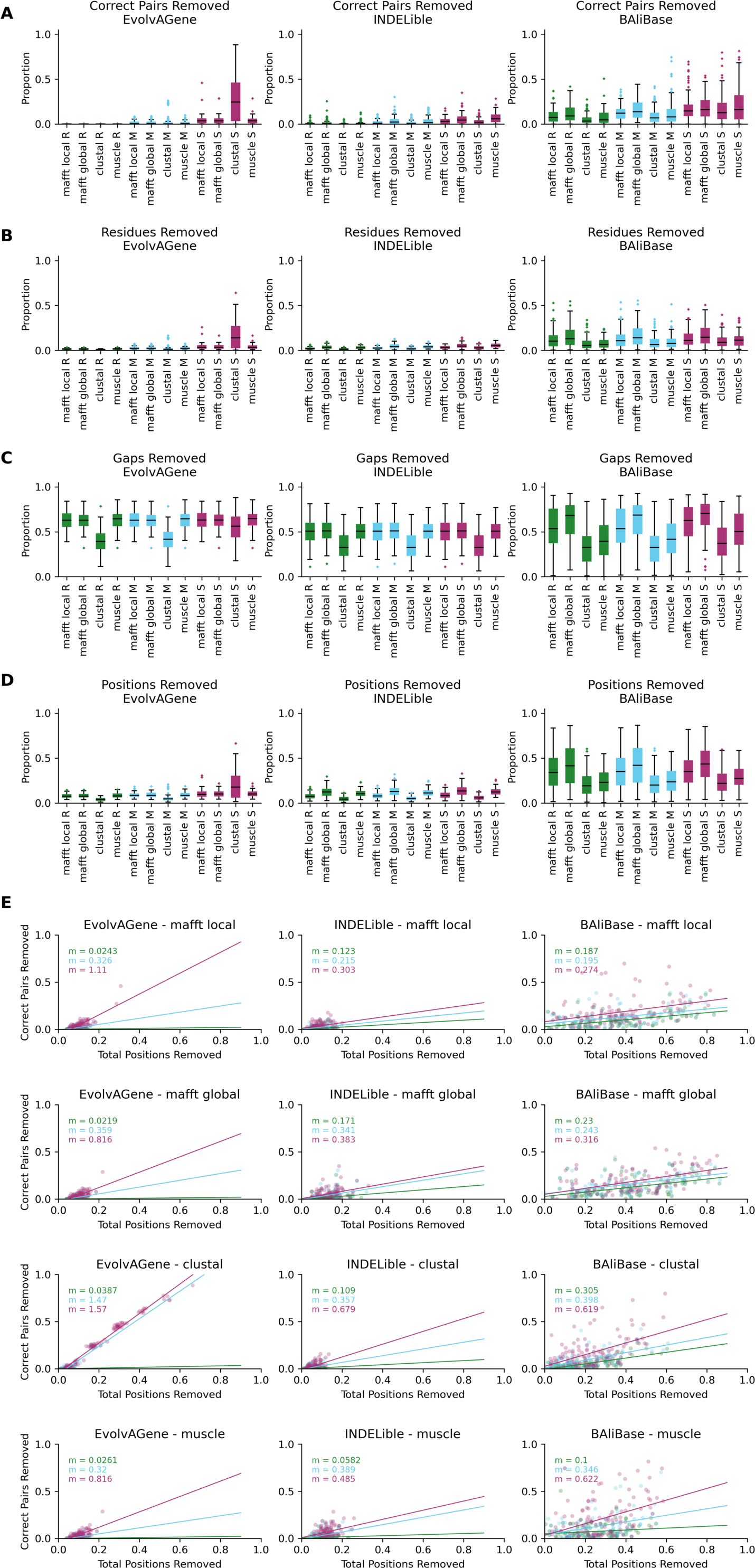

Supplement: Supplemental Information 2 — (A–D) Box plots showing the impact of running CIAlign cleaning functions with relaxed (green, R, left), moderate (blue, M, middle) and stringent (red, S, right) parameter values on alignments generated with MAFFT global (Katoh et al., 2002), MAFFT local (Katoh et al., 2002), MUSCLE (Edgar, 2004) and Clustal Omega (Sievers & Higgins, 2018) of sequences simulated using either EvolvAGene (Bahr et al., 2001) (left) or INDELible (Sievers & Higgins, 2018) (centre) and on the BAliBASE (Thompson, Plewniak & Poch, 1999) benchmark alignments (right), divided into: (A) proportion of correctly aligned pairs of residues (Sievers et al., 2013) removed (identified by comparison with a benchmark alignment), (B) proportion of total nucleotides (i.e. non-gap positions) removed, (C) proportion of gaps removed (D) proportion of positions (gap or non-gap) removed. (E) Scatter plots for each combination of simulation tool and alignment tool showing a linear regression analysis of the impact of the total proportion of positions removed on the proportion of correctly aligned pairs of residues removed by CIAlign for relaxed (green), moderate (blue) and stringent (red) parameter values. The statistic m is the slope of the regression line. [file peerj-10-12983-s002.png]

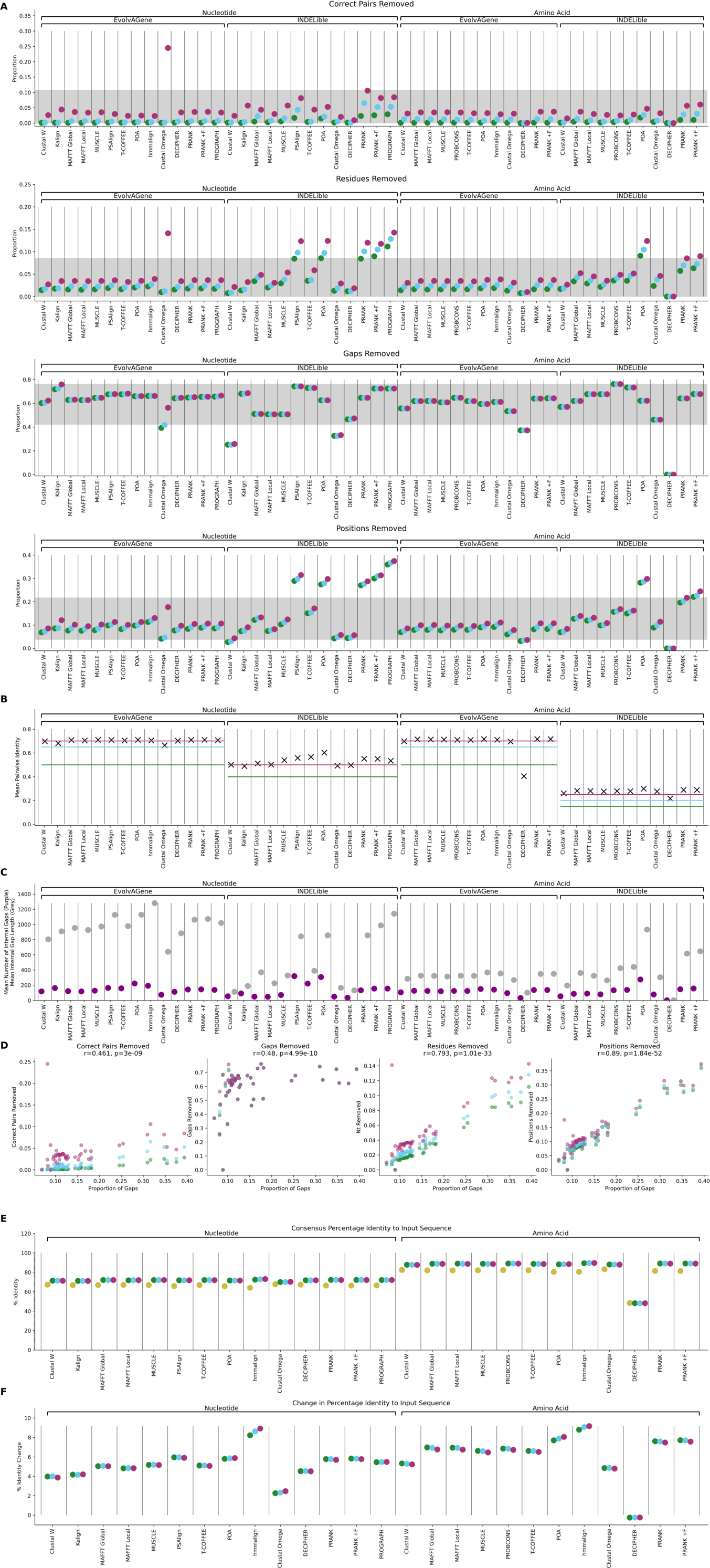

Supplement: Supplemental Information 3 — (A) Plots showing the impact of running CIAlign cleaning functions with relaxed (green, left), moderate (blue, middle) and stringent (red, right) parameter values on alignments of nucleotide and amino acid sequences simulated using either EvolvAGene (Bahr et al., 2001) or INDELible (Sievers & Higgins, 2018) and aligned with a large number of different alignment tools. From top to bottom, the y-axis represents proportion of correctly aligned pairs of residues (Sievers et al., 2013) removed (identified by comparison with a benchmark alignment), proportion of total nucleotides (i.e. non-gap positions) removed, proportion of gaps removed, proportion of positions (gap or non-gap) removed. (B) Plot showing the pairwise identity between sequences of nucleotides and amino acids in the alignments generated using different alignment tools and simulated using either EvolvAGene (Bahr et al., 2001) or INDELible (Sievers & Higgins, 2018), calculated as the mean of a similarity matrix generated with the CIAlign make_similarity_matrix_input function. Horizontal lines represent the threshold similarity used to remove divergent sequences under stringent (red), moderate (blue) and relaxed (green) CIAlign parameters for the different alignments (for INDELible nucleotide alignments relaxed and moderate are the same). (C) Plot showing the mean number of internal (non-terminal) gaps (purple) per alignment and the mean length of internal gaps (grey) for the alignments of nucleotides and amino acids generated using different alignment tools simulated using either EvolvAGene (Bahr et al., 2001) or INDELible (Sievers & Higgins, 2018). (D) Scatter plots showing the relationship between the metrics from (A) and the proportion of gaps in the alignment, r is the Spearman’s ρ correlation co-efficient and p the associated p-value. (E) Plots showing the mean percentage identity (top) and change in percentage identity (bottom) between consensus sequences generated from EvolvAGene (Bahr et al., 2001) [file peerj-10-12983-s003.png]

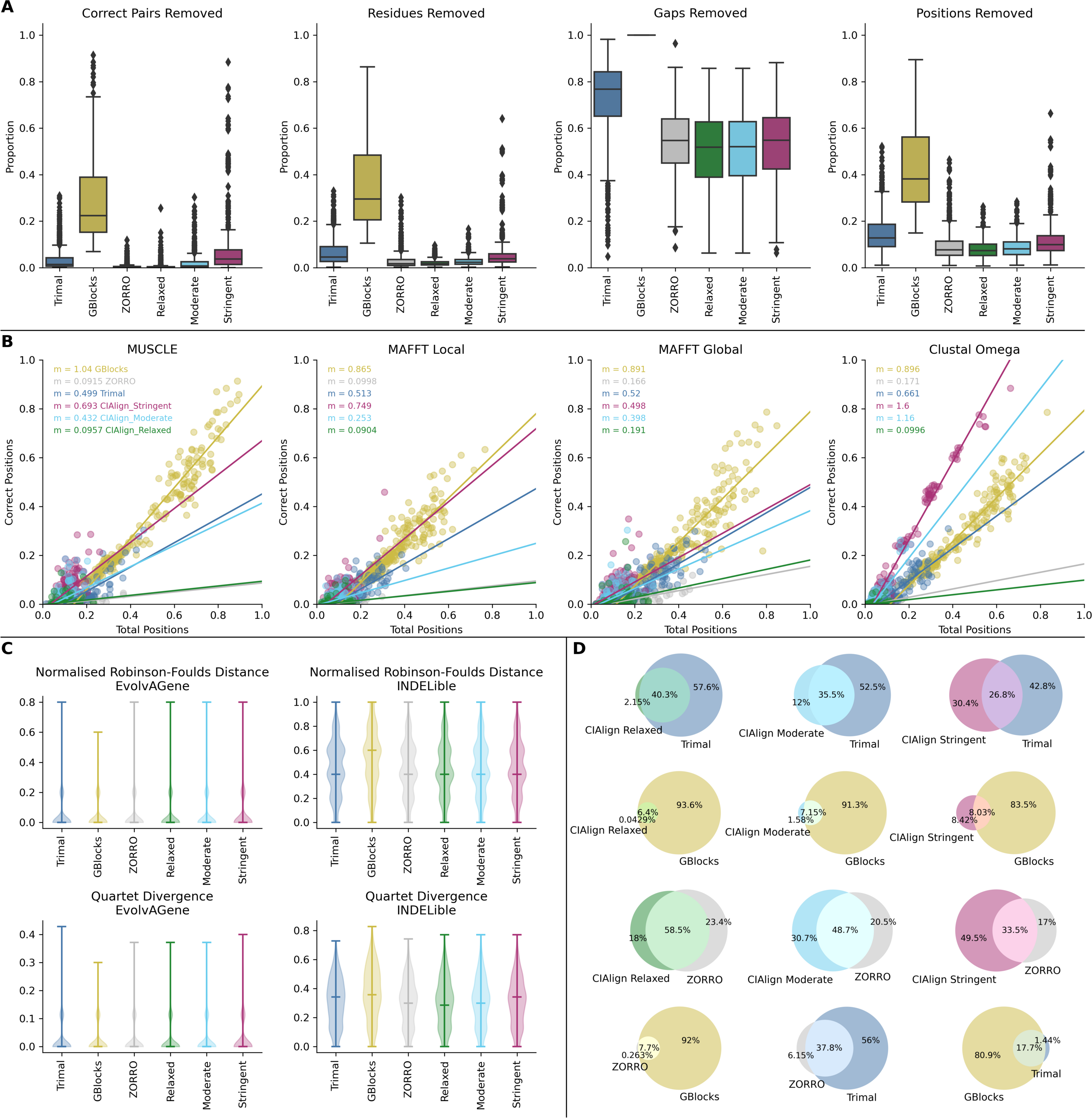

Supplement: Supplemental Information 4 — (A) Box plots showing the impact of running other trimming tools–TrimAl (cyan) (Capella-Gutiérrez, Silla-Martínez & Gabaldón, 2009), Gblocks (grey) (Talavera & Castresana, 2007) and ZORRO (orange) (Wu, Chatterji & Eisen, 2012) –and the CIAlign cleaning functions with relaxed (green), moderate (blue) and stringent (red) parameter values on alignments of sequences simulated using either EvolvAGene (Bahr et al., 2001) or INDELible (Sievers & Higgins, 2018). From left to right, the y-axis represents proportion of correctly aligned pairs of residues (Sievers et al., 2013) removed (identified by comparison with a benchmark alignment), proportion of total nucleotides (i.e. non-gap positions) removed, proportion of gaps removed, proportion of positions (gap or non-gap) removed. (B) Scatter plots showing linear regression analyses of the impact of the total proportion of positions removed after running CIAlign (stringent parameters, red; moderate parameters, blue; relaxed parameters, green), TrimAl (cyan), Gblocks (grey) and ZORRO (orange), on the proportion of correct positions removed by the tool for alignments generated with four alignment tools, from left to right MUSCLE (Edgar, 2004), MAFFT local (Katoh et al., 2002), MAFFT global (Katoh et al., 2002) and Clustal Omega (Sievers & Higgins, 2018). The statistic m is the slope of the regression line (C) Violin plots showing the distribution of normalised Robinson-Foulds distances (Hall, 2008) (left column) and Quartet divergence (right column) (Fletcher & Yang, 2009) between benchmark trees and test trees after running TrimAl, GBlocks and ZORRO versus CIAlign with the three sets of parameter values, for trees based on simulated sequences generated with EvolvAGene (Bahr et al., 2001) (top row) and INDELible (Sievers & Higgins, 2018) (bottom row). Red and black lines show the median and mean respectively. (D) Venn diagrams showing the overlap between aligned residue pairs (Sievers et al., 2013) removed when running CIAlign w [file peerj-10-12983-s004.png]

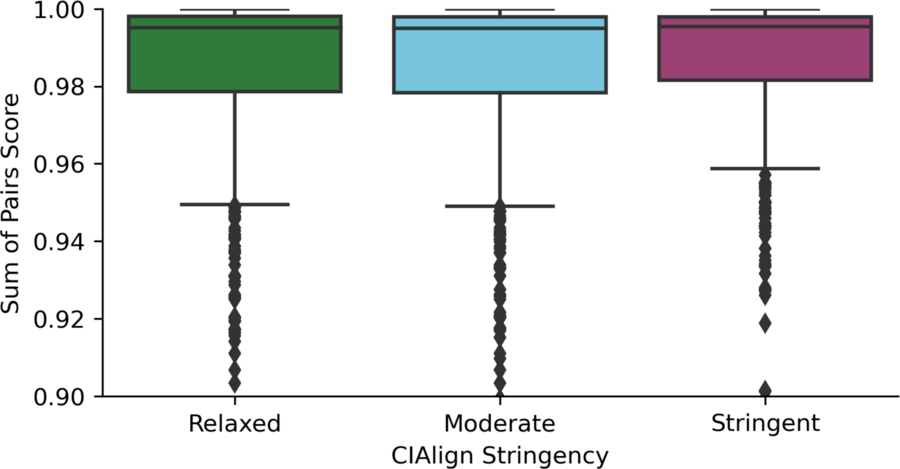

Supplement: Supplemental Information 5 — Box plot showing the distribution of the sum of pairs score (Sievers et al., 2013) comparing the CIAlign output with relaxed (left, green), moderate (middle, blue) and stringent (right, red) (Table S2) parameter settings and the same alignment after removing gaps and then re-aligning with the same software. [file peerj-10-12983-s005.png]

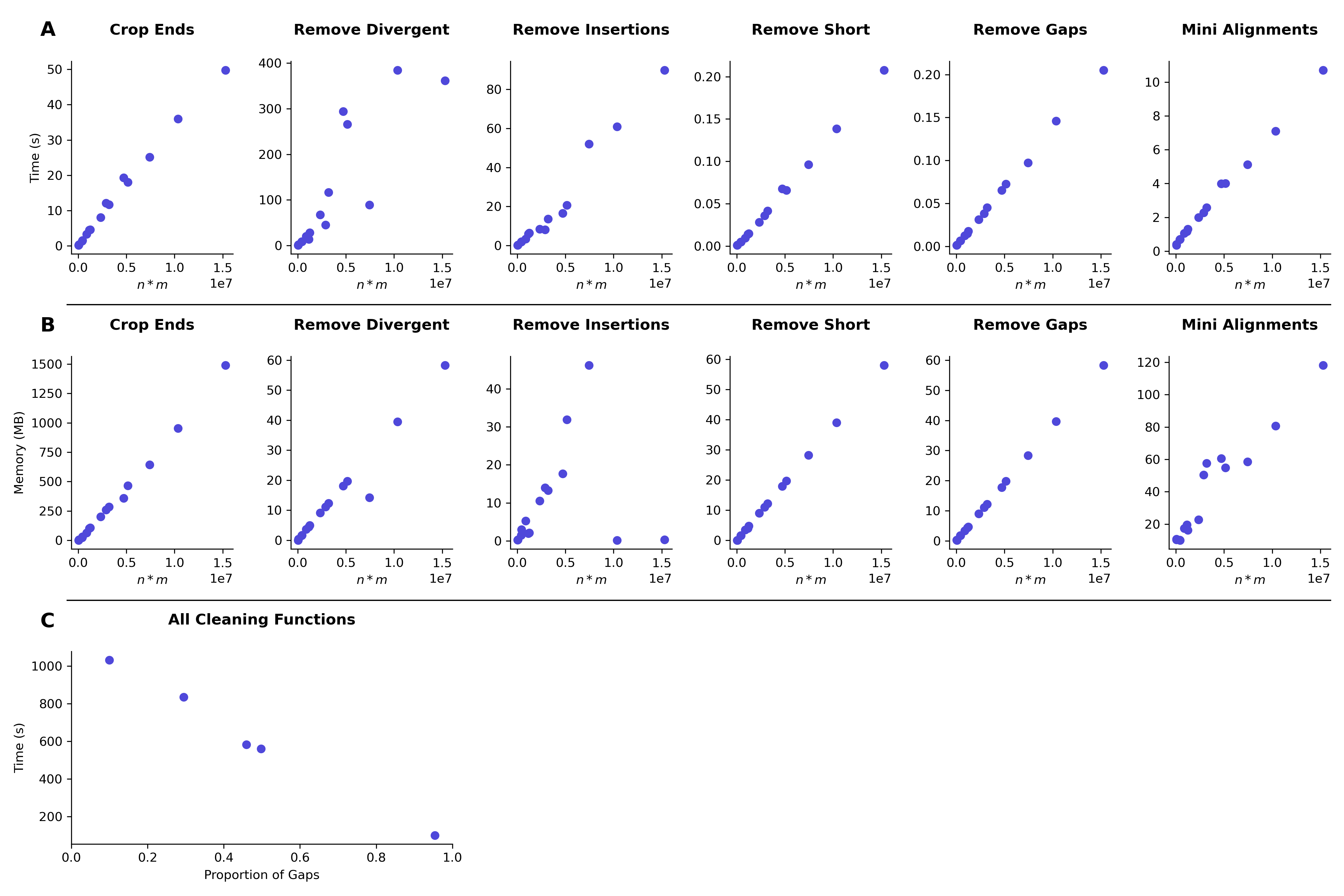

Supplement: Supplemental Information 6 — (A, B) Scatter plots showing the relationship between alignment size, measured as n (number of sequences) * m (number of columns) and the time (A) and memory (B) used by CIAlign to run each of the cleaning and mini alignment functions on the alignment, as the mean of four replicate measurements. 25 input alignments were chosen at random from the HomFam (Wright, 2015) benchmark set. All measurements were take n on one Intel Core i7-6700 core with 4 GB of RAM, running at 3.40 GHz. Memory was measured as resident set size. (C) The relationship between number of gaps in an alignment and the time taken to clean the alignment with CIAlign, measured by generating random MSAs of the same size (n = 2000, m = 3000) but with different proportions of gaps. [file peerj-10-12983-s006.png]

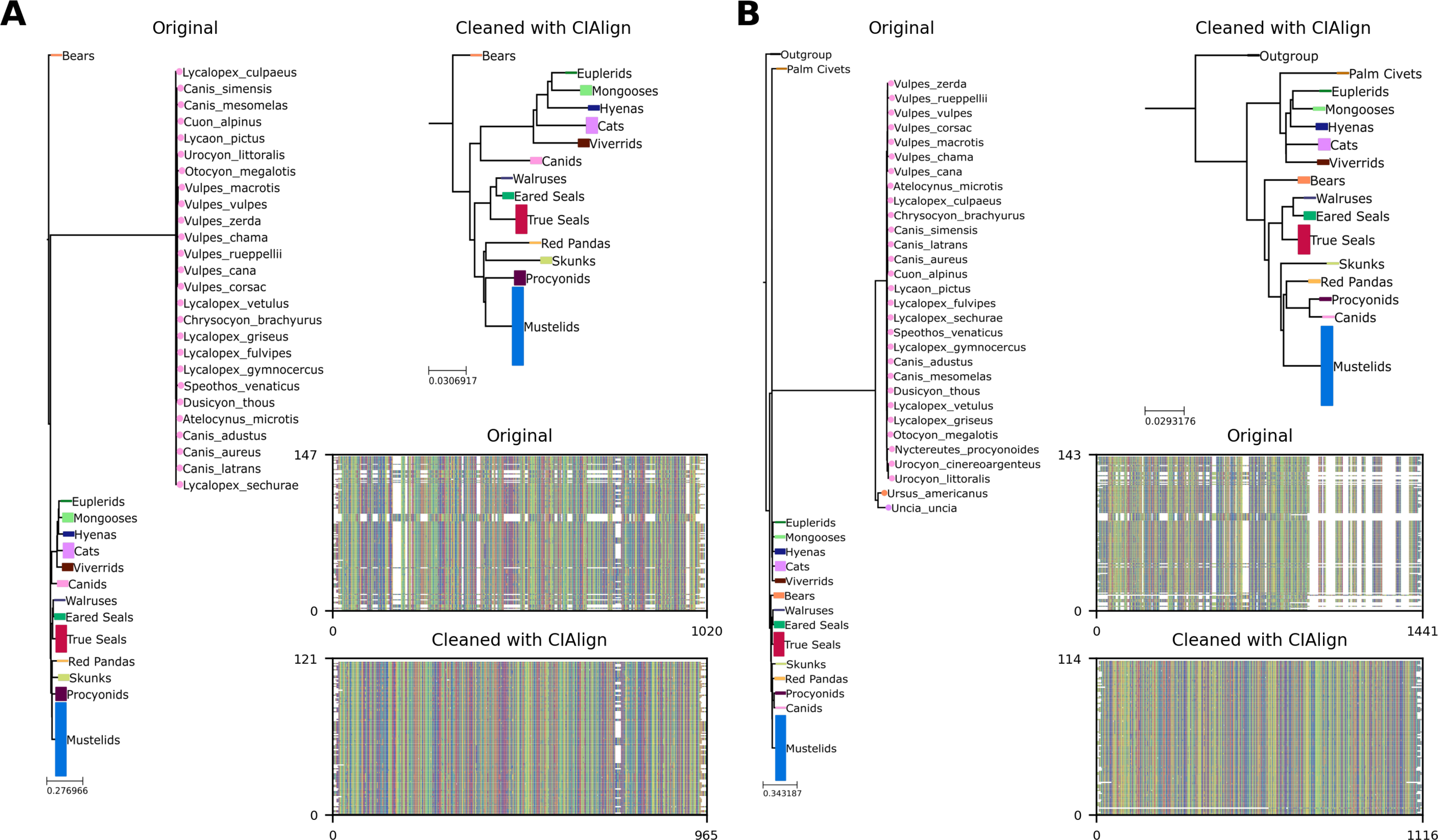

Supplement: Supplemental Information 7 — (A) Left, phylogenetic tree based on an alignment of sequences from the 10k trees project (Wick, 2019) for the APOB gene in Carnivora. Colours represent known monophyletic families of Carnivora. Nodes have been collapsed where multiple sequences from the same family formed a monophyletic clade. Sequences annotated with circles were removed by CIAlign. Top-right, tree based on the same alignment after cleaning with CIAlign, which removed the outlying group. Bottom-right, mini alignments showing the effect of running CIAlign on this alignment. (B) As for (A), but for the RAG1 gene in Carnivora. [file peerj-10-12983-s007.png]
