# Supplementary material for: CIAlign: A highly customisable command line tool to clean, interpret and visualise multiple sequence alignments
Source: PeerJ. 2022 Mar 15;10:e12983. doi: 10.7717/peerj.12983 (PMC8932311; doi:10.7717/peerj.12983)
Supplement: Supplemental Information 12 [file peerj-10-12983-s012.docx]

**Supplemental Materials and Methods**

**Benchmarking**

*Sequence Simulation*

Simulated sequences and alignments were generated using EvolveAGene4 [1], INDELible v1.0.3 [2] and BadRead v0.1.5 [3]. For EvolveAGene, 100 simulations were performed based on the human GAPDH coding sequence (Genbank accession: NC_000012.12, CCDS8549.1) with eight taxa, using the default settings and an average branch length of 0.62 (as recommended in the manual). For each simulation, this tool generated eight simulated coding sequences with associated true nucleotide and amino acid alignments and phylogenetic trees. For INDELible 100 simulations were performed using the ‘GTRexample’ parameters from the manual and the ‘nucleotide 1’ algorithm to simulate nucleotide evolution under the GTR model. The guide tree used and control files are available on the CIAlign GitHub. BadRead sequences were generated for 100 simulations each for the settings proposed for “very nice”, “mediocre” and “very bad” Oxford Nanopore reads in the BadRead documentation. All BadRead simulations were performed on the full length deformed wing virus reference sequence, Genbank Accession NC_004830.2. Shell scripts used to run these simulations are available in the benchmarking directory for each tool on the CIAlign GitHub as run_simulations.sh. The output data from all simulations is available at github.com/KatyBrown/benchmarking_data_CIAlign.

*BAliBase*

Along with the simulated alignments, the BAliBase [4] benchmark alignment database was used. All data was downloaded from the BaliBase web server (<http://www.lbgi.fr/balibase>) on 03/03/2021. 20 alignments (or all alignments for sets of less than 20) were selected at random from reference sets one to 10, the selected alignments are listed in Online Table 1. All gaps were removed using the CIAlign --unalign function prior to realignment.

*Alignment*

The EvolveAGene, INDELible and BAliBase sequences were aligned using Clustal (Omega version 1.2.4, [5]), with the default parameters plus --auto, MUSCLE (version 3.8.31, [6]), with the default parameters plus 100 iterations and MAFFT (version 7.464, [7]) with the default parameters (global) and with default parameters plus –localpair (local), both with 1000 iterations. BadRead sequences are expected to be incomplete, so were aligned only with MAFFT with the default parameters plus --localpair. Shell scripts used to generate these alignments are available in the benchmarking directory for each tool on the CIAlign GitHub as run_alignment.sh). All alignments are available at github.com/KatyBrown/benchmarking_data_CIAlign.

*HomFam Alignments*

The HomFam [8] benchmark alignment database was downloaded from <http://www.clustal.org/omega/homfam-20110613-25.tar.gz> on 05/07/2021. Sequences in the files labelled “_ref.vie” were used as seed sequences and sequences in the files labelled “_test-only.vie” as the test sequences, these were combined into single FASTA files. Alignments were generated using MAFFT (version 7.464 [6]) with the default parameters). CIAlign parameters are shown in Table S1. Trees were generated using FastTree2 with a GTR model (v2.1.10, [7]).

*CIAlign Cleaning*

CIAlign was used to clean all alignments with relaxed, moderate and high stringency parameters, parameter values are listed in Table S1. Generally the default parameters were used as the moderate parameter settings, the exception to this is remove_divergent, which was used at lower stringency for INDELible, BaliBase, BadRead and HomFam because these sequences are very divergent. Shell scripts used to run CIAlign are available in the benchmarking directory for each tool on the CIAlign GitHub as run_cialign.sh. All cleaned alignments are available at github.com/KatyBrown/benchmarking_data_CIAlign.

*Comparing Alignment Tools*

For Fig. S3 and Table S3, which compare a large number of alignment tools, the EvolvAGene and INDELible simulated nucleotide alignments described above were used. Simulated amino acid sequences and alignments are generated by default on running EvolvAGene. To generate simulated amino acid sequences with INDELible,100 simulations were performed using the ‘WAGexample’ parameters from the manual and the ‘amino acid 1’ algorithm to simulate amino acid evolution under the WAG model. All tools were used with both nucleotide and amino acid alignments unless designed specifically for only one or the other.

The following alignment tools and parameters were compared: Partial Order Alignment (POA) v2.0 [9] (nucleotide and amino acid) with default settings plus the NUC 4.4 and BLOSUM80 substitution matrices used by BLAST [10] for nucleotide and amino acid alignments respectively; Clustal Omega v. 1.2.4 [5] (nucleotide and amino acid) with the default parameters plus –auto, Clustal W v2.1 [11] (nucleotide and amino acid) with the default settings plus TYPE=DNA for nucleotide and TYPE=PROTEIN for amino acids; MUSCLE v3.8.31 [6] (nucleotide and amino acid) with the default parameters plus 100 iterations; MAFFT v 7.464 [7] with the default parameters for global alignments and with the default parameters plus –localpair for local alignments; T-COFFEE v11.0.8 [12] (nucleotide and amino acid) with the default parameters, PRANK v170427 [13] (nucleotide and amino acid) with the default parameters plus -DNA for nucleotide and the default parameters plus -protein for amino acids and with the same parameters plus “+F” (trust insertions) (labelled as PRANK and PRANK +F respectively), hmmalign v 3.3.2 [14] with the default parameters plus a profile HMM created with hmmbuild v3.3.2 [14] for the EvolvAGene input sequence (nucleotide and amino acid, EvolvAGene only); Kalign v3.2.2 [15] (nucleotide only) with the default parameters; DECIPHER v2.16.1 [16] (nucleotide and amino acid) with the default parameters via the AlignSeq command; PSAlign via psalign_tcoffee v1.10 [17] (nucleotide only) with the default parameters; PROGRAPH v20130709 [18] (nucleotide only) with the default parameters; PROBCONS v1.12 [19] (amino acid only) with the default parameters. Where appropriate, all outputs were converted to FASTA using the EMBOSS v6.6.0.0 [20] seqret function.

CIAlign parameters for all nucleotide alignments for relaxed, moderate and stringent settings were as shown in Table S1, amino acid alignments were much more divergent so the remove_divergent_minperc threshold was changed to 0.15, 0.20 and 0.25 for relaxed, moderate and stringent settings respectively for both EvolvAGene and INDELible, all other parameters remained the same.

*Comparisons*

Correctly aligned sequence pairs were identified by comparison with the benchmark alignments generated by the software and calculated using the get_POARs function implemented in the AlignmentStats module in the CIAlign benchmarking functions directory. Consensus sequences in all cases were generated in CIAlign with consensus type majority_nongap and phylogenetic trees with FastTree2 with a GTR model (v2.1.10, [21]). Identity and Needleman-Wunsch scores between pairs of sequences were calculated using the Needle tool from the EMBOSS package (v6.5.7.0, [20]). Robinson-Foulds distances were calculated using the compare function of the Python package ete3 (v3.1.1, [22]). Quartet divergence was calculated using the tqdist algorithm [23] implemented in the R package Quartet [24] [25], with similarity=FALSE to calculate divergence rather than similarity. All consensus sequences and trees are available at github.com/KatyBrown/benchmarking_data_CIAlign. All scores are available in Online Table 1 (for EvolvAGene, INDELible and BAliBase) and Online Table 2 (for BadRead) on the CIAlign GitHub in the benchmarking/tables directory. Column confidence scores were calculated with ZORRO version Linux_x86_64, with the default settings and a quality cutoff of 0.4 as suggested by the authors [26]. Linear regression was performed using the numpy polyfit function [27] with degree 1. For BadRead alignments, correctly aligned residues were identified by aligning the original input sequence to BadRead with the consensus sequence for the alignment used as the input to CIAlign. At positions where the consensus matched the input sequence, positions in the alignment which matched the consensus were then classified as correct.

*QuanTest2*

The impact of CIAlign on secondary structure prediction was tested using a modified version of QuanTest2 [28]. Alignments were created from the QuanTest2 “Test” datasets using MUSCLE (version 3.8.31, [6]) with the default settings. CIAlign parameters are shown in Table S1. Minor modifications were made to the QuanTest2 source code to adjust the indexing for columns removed by CIAlign. QuanTest2 by default removes all columns containing gaps in the reference sequence, however as CIAlign primarily acts on these columns the code was modified to instead use the whole alignment as the input to JPred4. The modified QuanTest2 script is available in the benchmarking/QuanTest2 directory of the CIAlign GitHub.

*Comparison with GBlocks, TrimAl and ZORRO*

CIAlign was benchmarked against three tools used to identify poor quality columns in MSAs, GBlocks [29], TrimAL [30] and ZORRO [26]. GBlocks v0.19d was used with the default settings plus -t=d to specify nucleotide sequences. TrimAL v1.4rev15 was used with the -automated1 setting to automatically detect the optimum method based on similarity statistics. ZORRO version Linux_x86_64 was used with the default settings and a quality cutoff of 0.4 was used as suggested by the authors [26]. All log files were converted to the same format as the CIAlign “removed” output files using the functions in convertLog.py, available on the CIAlign GitHub in the benchmarking/functions directory. All scores were then calculated as described above.

*Realignment*

For realignment, gaps were removed from the CIAlign output at the three stringency settings then the resulting FASTA file was realigned using the original alignment tool and parameters. Sum-of-pairs scores between the realigned MSA and the CIAlign output were calculated using the method described by Thompson et al. [4], implemented in the AlignmentStats.py module file available on the CIAlign GitHub in the benchmarking/functions directory.

**Biological Data**

Cleaning Pfam Alignments

A random sample of 500 Pfam domains was selected (listed in full in Online Table 8 in the benchmarking/tables directory on the CIAlign GitHub) and seed and full alignments for each domain were downloaded from Pfam release 34.0 [31]. All alignments were cleaned with CIAlign remove_insertions (insertion_min_size 1 and insertion_max_size 500) and crop_ends (crop_ends_mingap_perc 0.01, crop_ends_redefine_perc 0.1). Consensus sequences were generated in CIAlign with consensus type majority_nongap. 250 sequences were selected at random from each full alignment and aligned to consensus sequences from the seed and full alignments before and after CIAlign cleaning using the Needle tool from the EMBOSS package (v6.5.7.0, [20]), this tool was also using to calculate identity scores and Needleman-Wunsch scores.

*Removing Insertions and Deletions from Human Genes*

Human protein coding gene positions were identified based on human genome build GRCh38.p13 from Ensembl release 103 [32]. Genes were selected at random and single gene VCF files downloaded from the human genome project release 20170504 [33] via Tabix (v 1.1.9, [34]). Genes with at least one indel variant were selected until 25 insertions and 25 deletions (relative to the reference) had been identified, covering 32 genes in total. Individual genomes were then selected at random until a non-reference call for each variant had been identified in at least one individual. This process gave a set of 162 individuals. Separate VCF files were generated for each individual for each of the 32 genes and converted to FASTA files using bcftools (v1.9 [35]) with the -H A option to prioritise alternate variants. These FASTA files were aligned with MAFFT (version 7.464, [7]) with the default settings. They were then cleaned with CIAlign remove_insertions with insertion_min_size 1 and insertion_max_size 50. Full results including all gene Ensembl IDs, variant IDs and sample IDs are available in Online Table 9 on the CIAlign GitHub in the benchmarking/tables directory.

*Removing Outliers*

Unaligned single gene FASTA files were downloaded for mammalian genes from the 10k trees project [36] (version 3 for primates, version 1 for Carnivora). These files were aligned MAFFT (version 7.464, [7]) with the default settings. The alignments were then cleaned with the CIAlign remove insertions, crop ends, remove short and remove divergent functions, with default settings except for remove_divergent_minperc, which was set to 0.7 (as these are quite conserved sequences). Alignments before and after cleaning were visualised as CIAlign mini alignments with the default settings. Phylogenetic trees were generated for alignments before and after cleaning using FastTree2 with a GTR model (v2.1.10, [21]). Trees were pruned and labelled using the ete3 Python package (v3.1.1 [22]).

**References**

1. Hall BG. Simulating DNA coding sequence evolution with EvolveAGene 3. Mol Biol Evol. 2008;25:688–95.

2. Fletcher W, Yang Z. INDELible: a flexible simulator of biological sequence evolution. Mol Biol Evol. 2009;26:1879–88.

3. Wick RR. Badread: simulation of error-prone long reads. J Open Source Softw. 2019;4:1316.

4. Thompson JD, Plewniak F, Poch O. BAliBASE: a benchmark alignment database for the evaluation of multiple alignment programs. Bioinformatics. 1999;15:87–8.

5. Sievers F, Higgins DG. Clustal Omega for making accurate alignments of many protein sequences. Protein Sci. 2018;27:135–45.

6. Edgar RC. MUSCLE: a multiple sequence alignment method with reduced time and space complexity. BMC Bioinformatics. 2004;5:113.

7. Katoh K, Standley DM. MAFFT multiple sequence alignment software version 7: improvements in performance and usability. Mol Biol Evol. 2013;30:772–80.

8. Sievers F, Dineen D, Wilm A, Higgins DG. Making automated multiple alignments of very large numbers of protein sequences. Bioinformatics. 2013;29:989–95.

9. Lee C, Grasso C, Sharlow MF. Multiple sequence alignment using partial order graphs. Bioinformatics. 2002;18:452–64.

10. Camacho C, Coulouris G, Avagyan V, Ma N, Papadopoulos J, Bealer K, et al. BLAST+: architecture and applications. BMC Bioinformatics. 2009;10:421.

11. Larkin MA, Blackshields G, Brown NP, Chenna R, McGettigan PA, McWilliam H, et al. Clustal W and Clustal X version 2.0. Bioinformatics. 2007;23:2947–8.

12. Notredame C, Higgins DG, Heringa J. T-coffee: a novel method for fast and accurate multiple sequence alignment11Edited by J. Thornton. Journal of Molecular Biology. 2000;302:205–17.

13. Löytynoja A. Phylogeny-aware alignment with PRANK. Methods Mol Biol. 2014;1079:155–70.

14. Finn RD, Clements J, Eddy SR. HMMER web server: interactive sequence similarity searching. Nucleic Acids Research. 2011;39 suppl_2:W29–37.

15. Lassmann T, Sonnhammer EL. Kalign – an accurate and fast multiple sequence alignment algorithm. BMC Bioinformatics. 2005;6:298.

16. Wright ES. DECIPHER: harnessing local sequence context to improve protein multiple sequence alignment. BMC Bioinformatics. 2015;16:322.

17. Sze S-H, Lu Y, Yang Q. A Polynomial Time Solvable Formulation of Multiple Sequence Alignment. Journal of Computational Biology. 2006;13:309–19.

18. Szalkowski AM, Anisimova M. Graph-based modeling of tandem repeats improves global multiple sequence alignment. Nucleic Acids Research. 2013;41:e162–e162.

19. Do CB, Mahabhashyam MSP, Brudno M, Batzoglou S. ProbCons: Probabilistic consistency-based multiple sequence alignment. Genome Res. 2005;15:330–40.

20. Rice P, Longden I, Bleasby A. EMBOSS: the European Molecular Biology Open Software Suite. Trends Genet. 2000;16:276–7.

21. Price MN, Dehal PS, Arkin AP. FastTree 2--approximately maximum-likelihood trees for large alignments. PloS One. 2010;5:e9490.

22. Huerta-Cepas J, Serra F, Bork P. ETE 3: Reconstruction, Analysis, and Visualization of Phylogenomic Data. Mol Biol Evol. 2016;33:1635–8.

23. Sand A, Holt MK, Johansen J, Brodal GS, Mailund T, Pedersen CNS. tqDist: a library for computing the quartet and triplet distances between binary or general trees. Bioinformatics. 2014;30:2079–80.

24. Smith MR. Quartet: comparison of phylogenetic trees using quartet and split measures. 2019.

25. Smith MR. Bayesian and parsimony approaches reconstruct informative trees from simulated morphological datasets. Biol Lett. 2019;15:20180632.

26. Wu M, Chatterji S, Eisen JA. Accounting For Alignment Uncertainty in Phylogenomics. PLOS ONE. 2012;7:e30288.

27. Harris CR, Millman KJ, van der Walt SJ, Gommers R, Virtanen P, Cournapeau D, et al. Array programming with NumPy. Nature. 2020;585:357–62.

28. Sievers F, Higgins DG. QuanTest2: benchmarking multiple sequence alignments using secondary structure prediction. Bioinformatics. 2020;36:90–5.

29. Talavera G, Castresana J. Improvement of phylogenies after removing divergent and ambiguously aligned blocks from protein sequence alignments. Syst Biol. 2007;56:564–77.

30. Capella-Gutiérrez S, Silla-Martínez JM, Gabaldón T. trimAl: a tool for automated alignment trimming in large-scale phylogenetic analyses. Bioinformatics. 2009;25:1972–3.

31. Finn RD, Bateman A, Clements J, Coggill P, Eberhardt RY, Eddy SR, et al. Pfam: the protein families database. Nucleic Acids Res. 2014;42 Database issue:D222–30.

32. Yates AD, Achuthan P, Akanni W, Allen J, Allen J, Alvarez-Jarreta J, et al. Ensembl 2020. Nucleic Acids Res. 2020;48:D682–8.

33. Auton A, Abecasis GR, Altshuler DM, Durbin RM, Abecasis GR, Bentley DR, et al. A global reference for human genetic variation. Nature. 2015;526:68–74.

34. Li H. Tabix: fast retrieval of sequence features from generic TAB-delimited files. Bioinformatics. 2011;27:718–9.

35. Danecek P, Bonfield JK, Liddle J, Marshall J, Ohan V, Pollard MO, et al. Twelve years of SAMtools and BCFtools. GigaScience. 2021;10. doi:10.1093/gigascience/giab008.

36. Arnold C, Matthews LJ, Nunn CL. The 10kTrees website: A new online resource for primate phylogeny. Evol Anthropol Issues News Rev. 2010;19:114–8.

**Supplementary References Used for Example 4 (Figure S1)**

Fong JJ, Brown JM, Fujita MK, Boussau B. A Phylogenomic Approach to Vertebrate Phylogeny Supports a Turtle-Archosaur Affinity and a Possible Paraphyletic Lissamphibia. PLoS One. 2012;7. doi:10.1371/journal.pone.0048990.

Wolfe JM, Breinholt JW, Crandall KA, Lemmon AR, Lemmon EM, Timm LE, et al. A phylogenomic framework, evolutionary timeline and genomic resources for comparative studies of decapod crustaceans. Proceedings of the Royal Society B: Biological Sciences. 2019;286:20190079.

Almeida D, Maldonado E, Vasconcelos V, Antunes A. Adaptation of the Mitochondrial Genome in Cephalopods: Enhancing Proton Translocation Channels and the Subunit Interactions. PLOS ONE. 2015;10:e0135405.

Regier JC, Shultz JW, Zwick A, Hussey A, Ball B, Wetzer R, et al. Arthropod relationships revealed by phylogenomic analysis of nuclear protein-coding sequences. Nature. 2010;463:1079–83.

Hughes LC, Ortí G, Huang Y, Sun Y, Baldwin CC, Thompson AW, et al. Comprehensive phylogeny of ray-finned fishes (Actinopterygii) based on transcriptomic and genomic data. Proc Natl Acad Sci USA. 2018;115:6249–54.

Wiegmann BM, Trautwein MD, Winkler IS, Barr NB, Kim J-W, Lambkin C, et al. Episodic radiations in the fly tree of life. PNAS. 2011;108:5690–5.

Li H, Leavengood JM, Chapman EG, Burkhardt D, Song F, Jiang P, et al. Mitochondrial phylogenomics of Hemiptera reveals adaptive innovations driving the diversification of true bugs. Proc Biol Sci. 2017;284. doi:10.1098/rspb.2017.1223.

Misof B, Liu S, Meusemann K, Peters RS, Donath A, Mayer C, et al. Phylogenomics resolves the timing and pattern of insect evolution. Science. 2014;346:763–7.

Feng Y-J, Blackburn DC, Liang D, Hillis DM, Wake DB, Cannatella DC, et al. Phylogenomics reveals rapid, simultaneous diversification of three major clades of Gondwanan frogs at the Cretaceous–Paleogene boundary. PNAS. 2017;114:E5864–70.

Kawahara AY, Plotkin D, Espeland M, Meusemann K, Toussaint EFA, Donath A, et al. Phylogenomics reveals the evolutionary timing and pattern of butterflies and moths. PNAS. 2019;116:22657–63.

Laumer CE, Fernández R, Lemer S, Combosch D, Kocot KM, Riesgo A, et al. Revisiting metazoan phylogeny with genomic sampling of all phyla. Proceedings of the Royal Society B: Biological Sciences. 2019;286:20190831.

Garrison NL, Rodriguez J, Agnarsson I, Coddington JA, Griswold CE, Hamilton CA, et al. Spider phylogenomics: untangling the Spider Tree of Life. PeerJ. 2016;4:e1719.

Gu X-B, Liu G-H, Song H-Q, Liu T-Y, Yang G-Y, Zhu X-Q. The complete mitochondrial genome of the scab mite Psoroptes cuniculi (Arthropoda: Arachnida) provides insights into Acari phylogeny. Parasites & Vectors. 2014;7:340.

McKenna DD, Shin S, Ahrens D, Balke M, Beza-Beza C, Clarke DJ, et al. The evolution and genomic basis of beetle diversity. PNAS. 2019;116:24729–37.

Peters RS, Meyer B, Krogmann L, Borner J, Meusemann K, Schütte K, et al. The taming of an impossible child: a standardized all-in approach to the phylogeny of Hymenoptera using public database sequences. BMC Biology. 2011;9:55.
